# Editorial: Biofuels and Bioproducts From Anaerobic Processes: Anaerobic Membrane Bioreactors (AnMBRs)

**DOI:** 10.3389/fbioe.2021.694484

**Published:** 2021-06-02

**Authors:** Francesco Valentino, Paolo Pavan, Joan Dosta

**Affiliations:** ^1^Department of Environmental Sciences, Informatics and Statistics, Cà Foscari University of Venice, Mestre-Venice, Italy; ^2^Department of Chemical Engineering and Analytical Chemistry, University of Barcelona, Barcelona, Spain

**Keywords:** membrane bio reactors, bioproducts and biofuels, municipal wastewater, biogas, anaerobic bioprocesses

New biodegradable waste treatment configurations and technologies have arisen to support the transition of treatment plants toward resource recovery facilities. The interest in Anaerobic Membrane Biological Reactor (AnMBR) technology is increasing due to the advantages related to combine anaerobic digestion with membrane filtration. Thanks to the complete retention of anaerobic microorganisms, AnMBRs have the capacity to efficiently recover most of the energy potential in biodegradable waste streams in the form of biogas and produce high-quality effluents with low biomass production.

In both municipal and industrial wastewater treatment plants, a change of paradigm from aerobic treatment toward anaerobic treatment which uses the biodegradable organic carbon present in wastewaters to produce energy (as methane) and reduce the amount of sludge to be handled could find its practical application in AnMBR technology. Since the anaerobic treatment alone may not be enough to meet discharge limits and to hold low-rate anaerobic microorganisms, the utilisation of membrane technology coupled to anaerobic bioreactor is a promising solution to solve these issues. Fouling-related aspects are currently investigated since fouling mitigation can strongly contribute to energy saving, improving the economy for MBR-based wastewater treatment. In this perspective, different studies have been set up to address this issue, utilizing various reactor configurations and/or operating conditions. Other aspects to consider for the implementation of AnMBR are the effect of sulphate concentration in the influent wastewater that could be transformed to sulphide by sulphate-reducing bacteria while oxidizing biodegradable COD to CO_2_, the recovery of dissolved methane in the effluent, especially when working at psychrophilic temperatures, and the lack of nutrient removal in the treated effluent, among many others.

When applied for organic waste streams treatment, AnMBRs also could offer several advantages when compared to conventional anaerobic digestion treatment, including the footprint reduction thanks to the effective decoupling of hydraulic retention time (HRT) and sludge retention time (SRT), while achieving efficient hydrolysis and methanogenesis processes. In this scenario, AnMBRs application is still challenging, especially for those cases in which the waste substrate is characterised by a high suspended solids content and salinity, possible presence of toxic compounds and a variable influent composition.

In this Research Topic issue, the gathered contributions report successful applications of the AnMBR technology for the treatment of a wide variety of wastes and wastewaters. Depending on the studied case, the researchers selected suitable AnMBR configurations, Organic loading Rates (OLRs), initial inoculum and operating temperature ranges, among other parameters, yielding high COD removal efficiencies to produce methane-rich biogas and a high-quality effluent with reduced sludge production. Moreover, the published papers also take into consideration strategies devoted to alleviating membrane fouling and prevent flux decline, the modelling of the process performance and/or the study of the dynamics of the developed microbial communities under different operational conditions to better understand the biological process and the complex interactions of anaerobic microorganisms.

For the treatment of low strength municipal wastewater under psychrophilic temperatures, Ribera-Pi et al. analysed the performance of three upflow anaerobic sludge blanket (UASB) reactors, using different reactor configurations and inocula (namely, an UASB inoculated with flocculent biomass and two UASB-type AnMBRs, equipped with submerged hollow fiber membranes and inoculated with flocculent and granular biomass, respectively). In this study, the application of AnMBR technology led to a higher effluent quality in terms of soluble and particulate COD, BOD_5_ and TSS. Furthermore, under the conditions tested in AnMBRs, a slightly higher hydrolysis rates were recorded when flocculent biomass was used. These authors also highlighted the crucial need of dissolved CH_4_ recovery when anaerobic digestion is applied at low temperature conditions.

A sustainable treatment of concentrated industrial wastewaters could also be reached by means of AnMBR technology as reported by Muñoz Sierra et al., who studied the performance of an AnMBR equipped with a sidestream tubular membrane working at 55°C for the treatment of a high strength wastewater characterised by a phenol concentration up to 0.8 g phenol L^−1^ and a high salinity (18 g Na^+^ L^−1^). In this study, COD and phenol removal efficiencies of about 95% during long-term operation were recorded when the phenol loading rate did not exceed 20 mg phenol g^−1^ VSS d^−1^, which demonstrates the capability of AnMBR technology to convert organic pollutants into renewable methane energy under high saline conditions.

As explained above, the AnMBR technology could also offer several advantages for the treatment of biodegradable organic wastes when compared to conventional anaerobic digestion treatment. Within this framework, Ariunbaatar et al. studied the application of an AnMBR consisting of an UASB coupled with 2 side-stream tubular membrane modules connected in parallel to treat diluted FW under mesophilic conditions reducing the HRT successfully from the typical 20 d to 1 d. These authors recorded a COD conversion to biomethane up to 76% and the effect of membrane filtration contributed to obtain an overall percentage of COD removal above 97%.

Moreover, the study carried out by Iskander et al. demonstrates that codigestion of food waste with a suitable co-substrate (such as fats, oils and grease, FOG) could be successfully implemented in AnMBRs in both a single phase and a two-phase anaerobic digestion process equipped with a submerged flat-sheet ceramic membrane under mesophilic conditions, obtaining COD removal efficiencies above 98% attributed to both organics degradation and membrane separation.

The application of AnMBR technology to further valorise the liquid fraction from anaerobically digested organic wastes is also covered in this special issue topic by the study of Giménez-Lorang et al., which is focused on the treatment of the liquid fraction of a dry OFMSW anaerobic digestion effluent using a mesophilic AnMBR equipped with external tubular membranes. In this study, the AnMBR operation leads to high total and soluble COD removal efficiencies up to 38.5 and 70.4%, respectively, to produce a CH_4_ rich biogas. These authors observed an inhibitory effect on methanogenic biomass attributed to the high ammonia nitrogen content (3.6 ± 1.0 g NH_4_^+^-N L^−1^), that decreased at high SRT or low OLR.

Taken together, these papers present an overview of the high reliability, flexibility, and compactness that the AnMBR technology could bring to valorise a great variety of organic wastes and wastewaters to boost circular economy and provides an insight on the exciting research that is being performed to overcome the limitations of AnMBRs in order to fulfil the widespread application potential of this technology.

## Author Contributions

FV: supervision, writing—original draft, and project administration. PP: visualization. JD: writing—original draft, project administration, and conceptualization. All authors contributed to the article and approved the submitted version.

## Conflict of Interest

The authors declare that the research was conducted in the absence of any commercial or financial relationships that could be construed as a potential conflict of interest.

